# Feasibility analysis of conducting observational studies with the electronic health record

**DOI:** 10.1186/s12911-019-0939-0

**Published:** 2019-10-28

**Authors:** Marcel von Lucadou, Thomas Ganslandt, Hans-Ulrich Prokosch, Dennis Toddenroth

**Affiliations:** 1 Chair of Medical Informatics, Friedrich-Alexander-Universität Erlangen-Nürnberg, Erlangen, Germany; 2 Department of Biomedical Informatics, Mannheim University Medicine, Ruprecht-Karls-University Heidelberg, Mannheim, Germany

**Keywords:** Observational study, Retrospective study, Electronic health record, Data quality, Availability, Completeness, Currency, Granularity, Correctness

## Abstract

**Background:**

The secondary use of electronic health records (EHRs) promises to facilitate medical research. We reviewed general data requirements in observational studies and analyzed the feasibility of conducting observational studies with structured EHR data, in particular diagnosis and procedure codes.

**Methods:**

After reviewing published observational studies from the University Hospital of Erlangen for general data requirements, we identified three different study populations for the feasibility analysis with eligibility criteria from three exemplary observational studies. For each study population, we evaluated the availability of relevant patient characteristics in our EHR, including outcome and exposure variables. To assess data quality, we computed distributions of relevant patient characteristics from the available structured EHR data and compared them to those of the original studies. We implemented computed phenotypes for patient characteristics where necessary. In random samples, we evaluated how well structured patient characteristics agreed with a gold standard from manually interpreted free texts. We categorized our findings using the four data quality dimensions “completeness”, “correctness”, “currency” and “granularity”.

**Results:**

Reviewing general data requirements, we found that some investigators supplement routine data with questionnaires, interviews and follow-up examinations. We included 847 subjects in the feasibility analysis (Study 1 *n* = 411, Study 2 *n* = 423, Study 3 *n* = 13). All eligibility criteria from two studies were available in structured data, while one study required computed phenotypes in eligibility criteria. In one study, we found that all necessary patient characteristics were documented at least once in either structured or unstructured data. In another study, all exposure and outcome variables were available in structured data, while in the other one unstructured data had to be consulted. The comparison of patient characteristics distributions, as computed from structured data, with those from the original study yielded similar distributions as well as indications of underreporting. We observed violations in all four data quality dimensions.

**Conclusions:**

While we found relevant patient characteristics available in structured EHR data, data quality problems may entail that it remains a case-by-case decision whether diagnosis and procedure codes are sufficient to underpin observational studies. Free-text data or subsequently supplementary study data may be important to complement a comprehensive patient history.

## Background

Medical research provides evidence to support clinical decisions through experimental and observational studies [1, 2]. In experimental studies, the investigator determines who is exposed to interventions such as novel treatment options, whereas in observational studies the investigator can only observe the effect of an intervention and does not exert influence on patient exposures. Both of these study types, however, examine potential associations as indicators of causal relationships between exposures and outcomes. Exposures can be any intervention or circumstance which potentially affects the subsequent clinical course, from the administration of a certain medication to environmental factors. The outcome, in contrast, stands for any event or patient feature that the exposure may influence, such as subsequent complications, clinical recovery, or mortality. Experimental studies are believed to yield the highest level of evidence, although both study types contribute to the medical decision-making [3], and the question of which study type tends to be more credible can be debated [4, 5]. The initiation and completion of experimental studies is complex and expensive, and still may not be appropriate to all research questions due to practical and ethical issues, such as those found in the context of testing medications for embryotoxicity during pregnancy or in surgical studies.

Observational studies may use a retrospective design, where study data are acquired from existing past data sources or recall of earlier events [6], while a future collection and analysis of study data according to earlier plans is termed prospective.

Nowadays substantial amounts of clinical data available in electronic health records (EHRs) have accumulated during patient care, and can in principle be unlocked for research and used in observational studies. The growing availability and accessibility of EHR data promises to promote novel research opportunities [7], driven by the potential of efficiently including considerable study populations. Previous efforts to reuse EHR data have primarily targeted patient recruitment for experimental studies [8]. Abundant EHR data, however, may also facilitate the computerization of retrospective observational studies by minimizing the time and cost required to gather study data [9, 10]; data thus collected may be suitable *for “complementing the knowledge gained from traditional clinical trials”* [11].

In addition to evolving retrospective observational studies by utilizing EHR data, digital patient data could also support innovative approaches such as linking multiple databases from different health care providers in order to illustrate treatment pathways [12], or using genetic data. Further, the analysis of data that originate from the process of delivering clinical care, such as the frequency with which laboratory tests are ordered, may in the future also deliver information about patients’ health status [13].

Despite these promising opportunities in the scientific reuse of patient data in the EHRs, several studies have encountered data quality issues such as incompleteness, inaccuracy and inconsistency [14, 15]. Approaches to overcoming these challenges may vary due to differences in organizational structure and medical documentation between hospitals or health care systems [16]. These intricacies currently represent obstacles to the full practical utility of employing EHR data in observational studies.

The main objectives of this study are to:

1. Assess the general data requirements of observational studies
2. Analyze the feasibility of conducting observational studies with structured EHR data, in particular diagnosis and procedure codes

## Methods

### Selection of observational studies and assessment of general data requirements

The university hospital in Erlangen comprises 24 clinical departments and seven research institutes. We selected three departments and performed a MEDLINE search on their published observational studies. To cover a wide range of medical research topics, we chose the departments of Plastic and Hand Surgery (DPS), Gastroenterology (DG) and Radiation Oncology (DRO). As the Medical Subject Headings (MeSH) term “Observational Studies” was not introduced until 2014, we used the term “retrospective studies” to obtain studies published prior to this date. In order to also capture recent publications from the three departments, we limited our search to publications from the last 10 years whose authors included the current department heads. This query returned a total of 38 studies. We excluded studies to which we could not obtain full text access (*n* = 1), whose corresponding author appeared to belong to a different department or university (*n* = 22), or whose study population was recruited at a different university (n = 1). This procedure aimed to ensure that the study populations have similar exposures, for instance in terms of socioeconomic characteristics, and that some study data will likely have originated from routine documentation processes conducted within our local organizational structure, reducing the potential impact of database heterogeneity [17].

**Fig. 1:**
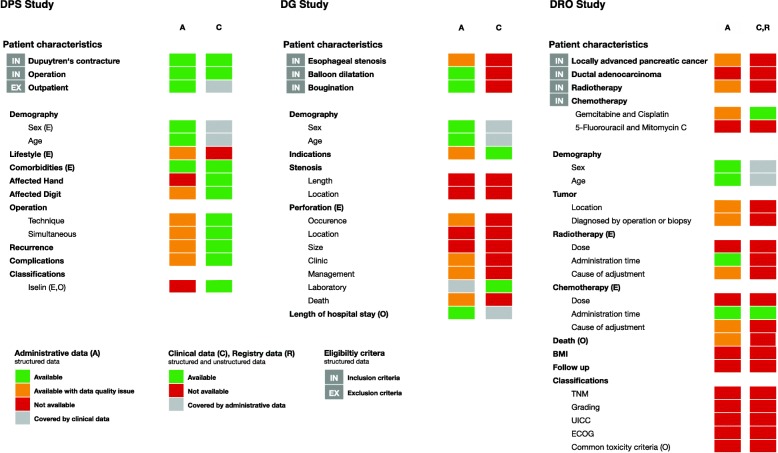
Overview of available eligibility criteria and other patient characteristics. A patient characteristic was considered available if it had been documented at least once. A detailed overview of exposure (= E) and outcome (= O) variables can be overviewed in detail in Table 1

We manually reviewed abstracts and methods sections of the remaining publications (*n* = 14) for general data requirements. For the feasibility analysis we randomly selected one of these studies from each of the three departments [18–20]. To delineate needed data, we extracted the eligibility criteria and other evaluated patient characteristics from the three selected publications, including exposure and outcome variables. To analyze time-dependent data requirements in each study and the involvement of various medical departments, we visualized the patient characteristics in a chronological sequence (see Additional file 1, pp. 4–6). We extracted patient characteristics from the text, tables and graphs of every publication and clustered these in semantic categories (see Fig. 1), and determined all exposure and outcome variables for each study (see Table 1).

**Table 1:** Study descriptions and detailed overview of investigated outcome and exposure variables from Fig. 1

1. DPS study	Exposure		A	C	Outcome		A	C
Assessing the relationship between typical risk factors and the severity of Dupuytren’s contracture *N* = 411								
	Comorbidities	Epilepsy Diabetes mellitus	X	–	Classifications	Iselin	–	X
	Lifestyle	Alcohol Smoking	X	–				
	Demography	Sex	X	–	Classifications	Iselin at initial or recurrent diagnosis of Dupuytren’s contracture	–	X
					Demography	Mean age at initial or recurrent diagnosis of Dupuytren’s contracture	X	–
	Classifications	Iselin (stage of Dupuytren’s contracture)	–	X	Complications	Postoperative Intraoperative	X	X
2. DG study	Exposure		A	C	Outcome		A	C
Perforation numbers after endoscopic esophageal balloon dilatation and bougination N = 423	Perforation	Perforation No Perforation	X	–	Length of hospital stay		X	–
3. DRO study	Exposure		A	C/R	Outcome		A	C/R
Comparison of two radiochemotherapies protocols in locally advanced pancreatic cancer N = 13	Radiotherapy Chemotherapy	Radiotherapy + Gemcitabine and cisplatin	X	X	Classifications	Common toxicity criteria	–	–
		Radiotherapy + 5-Fluororuacil and mitomycin C	–	–	Death		X	–
	Additive chemotherapy		X	X	Death		X	–

“X” = available, “-” = unavailable, A: Administrative data (structured data), C: Clinical data (structured and unstructured data), R: Registry data (structured data)

### Data definitions; overview of available EHR data

Our studied EHR aggregates heterogeneous medical data from various information systems at the university hospital in Erlangen [21]. According to original documentation purposes, we differentiated between three general data sources within our EHR:

1. Administrative data, which consists of hospital reimbursement data based on Diagnosis Related Groups (DRGs), using diagnostic codes from the International Classification of Diseases (ICD) and procedure codes from the “Operationen- und Prozedurenschlüssel” (OPS), which is the German modification of the International Classification of Procedures in Medicine (ICPM)
2. Clinical data, which contains routine data for medical decision-making and communication (e.g. clinical notes, discharge summary, results of laboratory tests)
3. Registry data, which has been documented by clinicians or trained data managers for clinical studies or epidemiological surveillance (e.g. cancer registry data).

We considered the latter two as a relative gold standard [22] as they are deemed to be more reliable, insofar as both are allegedly independent from reimbursement-associated incentives and the greater diligence [23–26] characterizing their documentation. Further, the view is that free text from clinical data is more flexible and expressive than coded data [27].

We additionally distinguished between two data categories in these data sources: (i) structured data, which contains coded data elements in accordance with standardized terminologies (e.g. ICD, OPS), and (ii) unstructured data, which consists of uncoded data elements like free-texts such as discharges summaries. The scientific usage of these categories differs in that free text may require structuring and harmonization prior to statistical interpretation for an observational study.

### Mapping of eligibility criteria onto structured EHR data

As only part of the available information systems at the university hospital in Erlangen have been incorporated into the EHR to date, our current recruitment options are mostly limited to diagnosis and procedure codes from administrative data. Consequently, the eligibility criteria from the publications had to be formalized in the corresponding diagnosis and procedure codes to identify eligible patients in our EHR. The occasional complexity and ambiguity of diagnosis and procedure codes means that eligibility criteria cannot always be unequivocally encoded [28]; further, documentation habits of coders may also vary [15, 29], so that in some cases choosing only a single code out of several coding possibilities is insufficient and can falsely exclude patients that should actually be eligible. Therefore, our more comprehensive approach to mapping ambiguous eligibility criteria was as follows:

1. Where the eligibility criteria do not permit assignment of a precisely corresponding code in the EHR data, we approximated its information with other structured data by applying computed phenotypes [30]. In the DRO study, for example, “locally advanced pancreatic cancer” could not be mapped to a closely agreeing diagnostic code. By definition, “locally advanced pancreatic cancer” is a surgically unresectable pancreatic cancer without distant metastasis [31], which we expressed by combining the diagnostic code for malignant pancreas tumor (C25) with M0, which is part of TNM classification for staging malignant tumors, and describes the absence of metastasis as determined via imaging and/or surgery [32]. As an alternative to this approach, we could have excluded cases with diagnostic codes for existing metastasis (C78 and C79).
2. If similar information could be derived from allocated diagnosis and procedure codes in the eligibility criteria, we used only the code with the larger number of patients as an eligibility criterion. In the DG study, for example, the procedure code for bougination and dilatation (5–429.7 and 5–429.8) implies a stenosis in the esophagus, although requiring another explicit diagnostic code for esophagus stenosis (K22.2) as the primary or secondary diagnosis would decrease the recruitment number. This inconsistency is due to differences in documentation habits and the availability of a variety of coding possibilities [28, 29, 33].
3. When unsure as to the adequate code allocation, we made use of the hierarchical structure of the coding system and assigned more general (superordinate) categories as eligibility criteria that were hypernyms of the respective codes of interest, in order to avoid falsely excluding eligible patients by applying overly specific criteria. As a result, we obtained a preliminary study population, for which we subsequently determined eligibility by manual evaluation of unstructured and structured data and assignment of a more specific code set, if possible. In the DRO study, for example, we initially included only patients who had received any chemotherapy or radiotherapy protocol.

If this approach did not enable us to identify available codes that would exactly match the published eligibility criteria, we still tried to approximate the intended study population with the available structured data by combining criteria by including and excluding codes or by applying computed phenotypes; the DRO study, in which we approximate the study population by the administration time of the chemotherapy, can serve as an example of this. Further, we determined a primary diagnosis in the eligibility criteria of the DG study to ensure that the specific diagnosis was treated in the encounter recorded.

MVL, a trained physician, carried out the mapping, of which an overview is included in the Additional file 1, pp. 7–9.

### Analysis of EHR data

For every eligible subject, all available structured and unstructured data were retrieved from our EHR and transferred into the table structure of the “i2b2” data analysis platform. A Comma-Separated Values (CSV) export was used for further analysis in the R statistics environment [34]. We created frequency tables of all structured data available in the study population. These tables present the various classification systems, such as ICD, OPS and Anatomical Therapeutic Chemical (ATC) classifications, in text form in their hierarchical structure, enabling an overview of categorical assignment of data. To produce a chronological overview of the clinical course of each patient, we generated individual visualizations of structured and unstructured data (patient files). All codes were automatically transformed into human-readable descriptions using the corresponding code libraries [35]. An eXtensible Markup Language (XML) export from R of the thereby assembled patient files and of frequency tables was transformed into hypertext using an eXtensible Stylesheet Language Transformation (XSLT) in order to make their content accessible in a web browser for further inspection (see Additional file 1, pp. 2–3). Where a study contained follow-up visits, we output all subsequent encounters after the initial diagnosis. The DRO study, for example, had a follow-up of 3 to 6 months, so all encounters after the first occurrence of the diagnosis of pancreatic cancer were output in the patient files.

These frequency tables and patient files allowed us to overview and identify potential documentation habits and relevant patient characteristics. The chronological order of unstructured and structured data in our patient files helped us to assess data quality and potentially refine eligibility criteria which initially had been mapped on unspecific codes or had been unavailable.

### Feasibility analysis

In the feasibility analysis, we first assessed the availability of relevant patient characteristics in the EHR for the three selected studies (see Fig. 1), and then evaluated the data quality of the available structured data for conducting observational studies by computing distributions of relevant patient characteristics and by comparing them to those from the original study. We categorized our findings according to four data quality dimensions and adopting the definitions proposed by Weiskopf et al. [36–38], and thus analyzed how well structured EHR data are “sufficient in quantity” (completeness), “free from error” (correctness), “recorded at the desired relative and absolute time(s)” (currency) and “neither too specific nor too broad” (granularity).

The assessment of granularity required expert opinion and determined the reliability of using certain structured data for computing distributions of patient characteristics. If available structured data did not conform to needed features, we attempted to revert to computed phenotypes to bypass the granularity issue. In the presence of insufficient granularity, we still used some available structured data in cases when we expected that the structured data would most likely provide the information we needed. For example, in the computed frequency of the patient characteristic “esophagus perforation caused by the procedure bougination or balloon dilatation”, we assumed that the diagnosis code for esophagus perforation, used in the same encounter as the procedures codes for bougination or balloon dilatation, would most likely be caused by either of these procedures than by other causes (such as vomiting).

We compared the patient characteristic distributions obtained to those from the original study, which we used as an external comparator for evaluation of completeness, assuming that a lower relative frequency might point to underreporting, i.e. incompleteness. We analyzed correctness in random samples by manually comparing the agreement between administrative data and a relative gold standard data source such as free text from histology reports or operation notes of clinical data. We evaluated currency by assessing how well the accompanied timestamps in our data reflected the clinical course.

## Results

The following section presents our findings in the context of our study’s two main objectives. We initially report on general data requirements in observational studies, and then cover the feasibility analysis of conducting observational studies with structured EHR data.

### General data requirements in observational studies

Besides routine data as the primary data source for observational studies, we found three of 14 manually reviewed studies to use additional data such as questionnaires, interviews and follow-up examinations to supplement their study data. We excluded these three studies from the random study selection process for the feasibility analysis, because these investigators’ individual data needs are not expected to be part of the EHR.

### Feasibility analysis

#### Data availability assessment

The university hospital in Erlangen has yet to incorporate all its information systems into the EHR; our assessment of data availability was therefore limited to the current state of the EHR at the time of our analysis. Figure 1 gives an overview of the available eligibility criteria and other patient characteristics of the three selected studies, clustered in semantic categories. The DPS study was the only one in which all patient characteristics were completely available in either administrative or clinical data, while in the other two studies some patient characteristics were unavailable.

Table 1 shows additional details of exposure and outcome variables. All exposure and outcome variables from the DG study were available in structured form, while in the DPS study we had to revert to unstructured data for some patient characteristics. In the DRO study, some variables were unavailable.

Over the course of the observational periods in the three selected studies, we found that some patient characteristics were documented in the EHR only from certain points in time on (an example here is ICD-O-3), or had been captured only up to a certain date (e.g. laboratory findings), and were thus incomplete over time (see Additional file 1, pp. 11–13).

In the DPS study, all three eligibility criteria from the publication were available in structured EHR data. We included 411 patients from November 1999 to September 2016 that met these criteria. In the DG study, all three eligibility criteria from the publication were available in structured EHR data. We identified 423 patients, treated between December 2000 and September 2015, using only the procedure codes for bougination and dilatation, which already imply a stenosis in the esophagus in their code description. Explicitly requiring the diagnostic code for esophagus stenosis (K22.2) as described in the publication had lowered the number of eligible subjects (*n* = 371). Since patients from the original publication had been treated only with either bougination or dilatation, we excluded 53 patients, who had received both interventions.

In the DRO study, 3 of 5 reported eligibility criteria were available in the structured EHR data, and data for one of two reported chemotherapies were available (gemcitabine and cisplatin from ATC codes). Due to the unavailability of histology and tumor stage, we attempted to identify eligible subjects by the administration time of the chemotherapy with a tolerated deviation of two days as described in the treatment protocol using the timestamps of the procedure codes, which seemed to match the administration times. We identified 13 subjects treated between January 2008 and May 2015 with similar chemotherapy protocols (see Additional file 1, p. 16).

#### Data quality assessment

Table 2 shows the distributions of patient characteristics, which we were able to compute with the available structured EHR data, in particular with diagnosis and procedure codes from administrative data. Each encountered contextual data quality issue, marked as orange boxes in Fig. 1, are discussed in detail in Table 3. It should be noted that the included study populations originated from different observational periods compared to the original study.

**Table 2:** Overview of the computed patient characteristic distributions from our EHR structured data in comparison to the original study

DPS study					
Patient characteristics			Publication (P)	EHR (E)	
			DP: 1956–2006 (50 years) *n* = 2919	DP: Nov 1999 – Sep 2016 (17 years) *n* = 411	
Demography	Sex	Male, n (%)	2579 (88.4)	327 (79.6)	
		Female, n (%)	340 (11.6)	84 (20.4)	
	Mean age	Male, years	57.62	61.39	
		Female, years	62.62	64.57	
Complications	Nerve injury, n (%)		108 (3.7)	3 (0.73)	
	Tendon injury, n (%)		5 (0.2)	2 (0.49)	
	Skin necrosis, n (%)		76 (2.6)	0 (0)	
	Infection, n (%)		94 (3.2)	1 (0.24)	
	Bleeding, n (%)		35 (1.2)	7 (1.7)	
Comorbidities	Diabetes mellitus, n (%)		306 (10.5)	25 (6.1)	
	Epilepsy, n (%)		39 (1.3)	5 (1.2)	
			DP: 1988–2006 (18 years) *n* = 1119	DP: Nov 1999 – Sep 2016 (17 years) n = 411	
Demography	Sex	Male, n (%)	977 (87.3)	327 (79.6)	
		Female, n (%)	142 (12.7)	84 (20.4)	
Affected digit/s	One digit, n (%)		505 (45.1)	100 (24.3)	
	More than one digit, n (%)		614 (54.9)	263 (64)	
Operation technique	Limited fasciectomy, n (%)		1061 (94.8)	5 (1.2)	
	Total fasciectomy, n (%)		58 (5.2)	0 (0)	
	Additional amputation, n (%)		13 (1.2)	2 (0.5)	
Recurrence, n (%)			145 (13)	67 (16.3)	
Lifestyle	Smoking, n (%)		185 (16.5)	11 (2.7)	
	Alcohol, n (%)		236 (21.1)	4 (1)	
	Both, n (%)		76 (6.8)	0 (0)	
Simultaneous operations	Carpal tunnel, n (%)		14 (1.3)	33 (8.0)	
	Trigger finger, n (%)		26 (2.3)	44 (10.7)	
DG study					
Patient characteristics			Publication (P)	EHR (E)	
			DP: Jan 2002 – Dec 2011 (9 years) *n* = 368	DP: Dec 2000 – Sep 2016 (15 years) n = 423	
Demography	Sex	Male, n (%)	269 (73.1)	297 (70.2)	
		Female, n (%)	99 (26.9)	126 (29.8)	
	Mean Age	Total, years (range)	61.3 (21–94)	62.5 (19–96)	
		Balloon Dilatation, years	61.8	57.7	
		Bougination, years	63.4	63.5	
Balloon dilatation, Bougination	Patient with balloon dilatation, n (%)		68 (18.5)	69 (16.3)	
	Patient with bougination, n (%)		300 (81.5)	354 (83.7)	
	Sessions of balloon dilatation, n		211	136	
	Sessions of bougination, n		1286	1281	
	Total sessions, n		1497 (4.07 per patient)	1417 (3.35 per patient)	
Perforation	Balloon dilatations, n		0	0	
	Bougination, n		8	5	
	Occurence, n		During first session: 4 During second session: 4	During first session: 3 During second session: 1 During later sessions: 1	
	Detection, n		During procedure: 4 After procedure: 4	No specification: 5	
	Rate per procedure, %		0.53	0.35	
	Rate per patient, %		2.17	1.18	
	Death, n		1	0	
Length of hospital stay	With perforation, days (range)		21.3 (9–41)	19.2 (4–36)	
	Without perforation, days (range)		6.8 (2–13)	9.1 (1–80)	
DRO Study					
Patient characteristics			Publication (P)	EHR (E)	
			DP: unknown Gemcitabine and cisplatin *n* = 58	DP: Jan 2008 – May 2015 Gemcitabine and cisplatin n = 13	
Demography	Sex	Male, n (%)	36 (62)	10 (76.9)	
		Female, n (%)	22 (38)	3 (23.1)	
	Mean Age, years		63	60.5	
Tumor	Location	Head, n (%)	40 (69)	6 (46.2)	3 (23.1) (no specification)
		Body, n (%)	9 (16)	1 (7.7)	
		Tail, n (%)	1 (2)	0 (0)	
		Head body, n (%)	5(9)	3 (23.1)	
		Body tail, n (%)	3 (5)		
	Diagnosed by	Biopsy, n (%)	41 (71)	1 (7.7)	
		Operation, n (%)	15 (29)	0 (0)	
Median Duration	Radiotherapy, days		42	42	
Survival	Alive at analysis, n		2	10	

Note that the DPS study included two observational periods. *DP* = data period

**Table 3:** Critical appraisal of the included study populations and discussion of contextual data quality issues in patient characteristics (see orange boxes, Fig. 1)

DPS study			
Issues in the study population			
To ensure the condition had been treated during the considered encounter, we only included subjects with a primary diagnosis of Dupuytren disease. The sensitivity of this approach may be limited insofar as some patients with another primary diagnosis also might have been eligible.			
Patient characteristics		DQ violation	Issue
Recurrence		Granularity	A label for a recurrence of Dupuytren’s contracture was not available. We therefore implemented a computed phenotype by considering the diagnosis of Dupuytren as a recurrence when documented during a subsequent encounter. This phenotype may be biased in certain circumstances, as neither the diagnosis nor the procedure codes specified the affected hand. Accordingly, we could not differentiate between a recurrence and a novel affection of the other hand. Further, the patient could have already undergone surgery in a different hospital, so that his seeming first encounter may in fact represent a recurrence.
Operation	Technique	Completeness Granularity	The actually corresponding procedure codes for the described operation techniques in the original study were not frequently used in our EHR, which instead employed different procedure codes; this suggests that documentation habits may have affected frequency estimates. We were unable to clearly ascertain which procedure codes represented treatment of conditions that had been documented via simultaneous diagnostic codes.
	Simultaneous	Granularity	We computed the distribution of simultaneous surgeries by counting the secondary diagnoses of carpal tunnel and trigger finger. The corresponding procedure codes described surgical techniques that could be applied to treat several different hand conditions, so that we could not determine whether these diagnoses had been treated during the encounter.
Complications		Currency Granularity	Counting complications would require interpretations of plausible temporal and causal relationships, which we were not always able to infer from observable codes. When a subject had received more than one intervention during an encounter, for example, it was difficult to determine which of the corresponding clinical events happened first and caused each other.
Lifestyle		Completeness Granularity	Information on lifestyle features such as alcohol and smoking was not frequently coded in our EHR. The diagnostic codes did not clarify whether, for example, a patient had started or stopped smoking. We noticed an inconsistent use of these codes throughout patient encounters, which can be interpreted as a changing diagnostic status or incompleteness (see Additional file 1, p. 15).
Affected digits		Completeness Granularity	There was no diagnostic code available that specified the number of affected digits. To bypass this problem, we computed a phenotype from procedure codes that had specified the number of digits operated on. The phenotype provided incomplete numbers, which may point to low utilization of the included procedure codes in our EHR.
DG study			
Issues in the study population			
–			
Eligibility criteria		DQ violation	Issue
Esophageal stenosis		Completeness	We noticed an inconsistent coding of esophagus stenosis. Adding the diagnostic code for esophagus stenosis to the eligibility criteria as described in the publication would decrease the study population size.
Patient characteristics		DQ violation	Issue
Indication		Granularity Correctness	In some instances, we were unable to determine and count the indications for bougination or dilatation, as the etiology of the treated esophagus stenosis formation was occasionally not well reflected in the codes. Some patients had multiple diagnoses that could be considered as a potential indication. According to free-text notes, some subjects developed a postsurgical or postradiation esophagus stenosis after tumor treatment, but had only a tumor diagnosis coded.
Perforation: Occurrence, clinic, management		Currency Granularity	The computed number of intervention-associated perforations might be subject to bias, insofar as we could not retrace whether the endoscopic procedure carried out on the esophagus preceded the complication, or whether the coded perforation was a remnant of earlier medical history. Further, we could not determine the subsequent management of perforations and the associated clinical picture, as the diagnostic codes and their time stamps did not permit us to extrapolate causal chains and temporal sequences.
Death		Granularity	We had data on inpatient death dates. Information on the cause of death was not available.
DRO study			
Issues in the study population			
We filtered for patients who received the exact same chemotherapy protocol, varying by a maximum of +/− 2 days, which excludes subjects with a delayed administration or subjects with additional chemotherapy after the treatment. Further, since the disease stage and tumor histology type was not reflected in the codes, we could not tell if the radiochemotherapy in our recruited study population had been administrated for the primary tumor, distant metastasis, or for a different tumor.			
Eligibility criteria		DQ violation	Issue
Locally advanced pancreatic cancer		Granularity	No specific diagnostic code was available for this tumor entity.
Radiotherapy		Granularity	No specific procedure code was available for the radiotherapy protocol.
Chemotherapy		Granularity	No specific procedure code was available for the chemotherapy protocol.
Patient characteristics		DQ violation	Issue
Tumor	Location	Correctness Granularity	We computed the distribution of tumor location distribution from coded tumor location of the first encounter. For some subjects, the tumor location remained unspecified at this point. Further, we noticed a changing tumor location in subsequent encounters (see Additional file 1, p. 17).
	Diagnosed by operation/biopsy	Completeness	Only patient data from the university hospital in Erlangen were available for our analysis. Consequently, operations or biopsies performed at other hospitals could not be included in our distribution computation.
Death		Completeness Granularity	We only had data on death dates of patients who had died in the hospital. Information on the cause of death was not available.
Radiochemotherapy: Cause of adjustment		Granularity Currency	The cause of radiochemotherapy adjustment required interpreting temporal and causal relationships which could not be inferred from the codes.

We evaluated “correctness” if a corresponding gold standard data source was available, and “completeness” if the corresponding patient characteristic distribution had been calculated. *DQ*= Data quality

In all three studies, the distributions of demographic features followed a similar pattern, although in the DPS study, fewer subjects were included despite similar observation periods. The relative frequency of comorbidities was comparable (diabetes mellitus in 10.5% of the Publication (P) population and 6.1% of the EHR (E) population; for epilepsy the figures are P 1.3% and E 1.2%). The phenotype for the number of affected fingers per subject yielded different results, which may point to an inconsistent use of the included procedure codes in the phenotype (“one affected digit”: P 45.1%; E 24.3%, “several affected digits”: P 54.9%; E 64%). The procedure codes, which are equivalent to the surgical techniques described in the publication, were not commonly used in our EHRs (“limited fasciectomy”: P 94.8%; E 1.2%, “total fasciectomy”: P 5.2%; E 0%). The relative frequency of amputation was similar (“amputation”: P 1.2%; E 0.5%). Our phenotype for recurrence provided comparable percentages (“recurrence”: P 13%; E 16.3%). The lifestyle features seemed to be highly underrepresented in comparison to the publication data (smoking: P: 16.5%; E: 2.7%, alcohol: P: 21.1%; E: 1%). The percentages of complications calculated were similar (“nerve injury”: P: 3.7%; E 0.73%, “tendon injury”: P: 0.2%; E: 0.49%, “skin necrosis”: P: 2.6%; E: 0%; “infection”: P: 3.2%; E: 0.24%; “bleeding”: P:1.2%; E: 1.7%). Simultaneous operations were considerably more frequent in the EHRs (“carpal tunnel”: P: 1.2%; E: 8.0%, “trigger finger”: P: 2.3%; E: 10.7%).

In the DG study, we observed similar relative frequencies of documented procedures in the EHRs (balloon dilatation: P: 18.5%; E: 16.3%, bougination: P: 81.5%; E: 83.7%), but proportionally fewer numbers of procedures and perforations in total than described in the publication, although we extended the observation period from 9 to 15 years. Despite the extended observational period only 55 additional subjects were included. The average length of the hospital stays demonstrated similar results (“with perforation”: P: 21.3 days, range 9–41 days; E: 19.2 days, range 4–36 days; “without perforation”: P: 6.8 days, range 2–13 days; E: 9.1 days, range 1–80 days).

In the DRO study, the subjects showed similar distributions for tumor location. The median duration of radiotherapy was comparable (“median duration”: P: 42 days; E: 42 days). We could obtain little information from the EHRs on how the tumor entity was diagnosed (“biopsy”: P: 71%; E: 7.7%; “operation”: P: 29%; E: 0%). Information on survival displayed a large discrepancy (“alive at analysis”: P 2; E 10).

Below we summarize data quality violations in the available structured EHR data, in particular diagnosis and procedure codes from administrative data, categorized according to the four data quality dimensions.

1. Completeness: A small number of patient characteristics (e.g. operations techniques, lifestyle) had an equivalent code available, which had not been frequently utilized in our EHRs. Although the data quality for the patient characteristic “comorbidities” in the study DPS was considered reasonably well, we noticed some inconsistent coding of the chronic diseases diabetes and epilepsy throughout the encounter history (see Additional file 1, p. 15). We additionally found procedure codes that would imply a certain diagnosis, which in practice had not been consistently documented, for example when esophageal bougination or balloon dilatation had been coded without an explicitly documented esophagus stenosis. Besides newly added diagnoses, of course, these phenomena could explain the varying number of diagnoses throughout the encounter history of some subjects (see Additional file 1, p. 14). Further, only patient data from our local hospital were available for analysis, which prohibited the inclusion of certain relevant features in the DRO study such as whether or when a patient died outside hospital, as well as diagnostic data from other hospitals to which a patient may have been transferred.
2. Correctness: Manual review of free-text operations and histology notes showed that on some occasions, the corresponding diagnostic codes intended to denote a suspected diagnosis. It seemed that suspected diagnoses from original descriptive free-text notes had sometimes been transformed into explicit diagnoses during coding. In the DG study, some pathology reports contained information about the indication for the endoscopic procedures which we could not derive from the corresponding diagnostic codes. For example, a postsurgical or postradiation esophagus stenosis after tumor treatment described in a pathology report has only been encoded as a tumor of the esophagus.
3. Currency: The time stamps of diagnostic codes appeared unsuitable for reliably inferring temporal details of individual clinical courses within an encounter, as most of the time the diagnostic codes share the same time stamps, although they in fact might have appeared at different times during the clinical course. On the other hand, the time stamps of procedures reproduced the sequence of procedures reasonably well (e.g. administration time of chemotherapies). In some instances, we observed diagnoses and procedures with the same time stamps, so that it is possible, to an extent, to presume a relationship between them in the sense that the procedure has likely been performed in order to treat the diagnosis. Typically, however, both codes appeared independently of each other. Further, a newly documented previous diagnosis could imitate a current diagnosis. Given the potential for temporal discrepancy and the possible interference of former diagnoses, such constellations could mask the actual clinical course.
4. Granularity: Some of the analyzed patient characteristics had only an unspecific code available or could not be unequivocally coded (e.g. locally advanced pancreatic cancer, number of affected digit). Sometimes we also noticed changes in the granularity of a documented diagnosis during the course of encounters; one instance of this in the DRO study was a change from an unspecified tumor location to a specific tumor location (see Additional file 1, p. 17). Occasionally, we observed that obvious causality (e.g. the cause of an esophagus perforation) and causal chains (e.g. the esophagus perforation that gave rise to a certain subsequent clinical management) are sometimes not well reflected in diagnosis and/or procedure codes with regard to which codes belong together or provoked each other. In addition, we sometimes could not determine whether a diagnosis relates to an acute, chronic, temporary, permanent, suspected, recurrent or resolved condition, which complicates the interpretation of the clinical course. For diagnoses that typically occurred concomitantly with certain associated procedure codes, it could be difficult to determine whether a disease was treated within the same encounter or on a different occasion.

## Discussion

This study evaluated general data requirements in observational studies and the feasibility of conducting observational studies with structured EHR data. While most data requirements of observational studies were fulfilled with routine data, we also found that some observational studies required additional data, which have been subsequently acquired. The differences in availability and data quality of diagnostic and procedure codes from administrative data impaired the feasibility of observational studies with structured data. Unstructured data such as free text need to be incorporated to complement gaps in structured data and receive valid patient characteristics.

Our findings on subsequently acquired study data with questionnaires, interviews and follow-up examinations underline the specific requirements from investigators, which cannot be found in the EHR and would necessitate contacting former patients for an observational study.

The data availability assessment of the patient characteristics from the three selected observational studies provided a good overview of the current state of our EHR. If we look at the entire EHR, we found all patient characteristics from the DPS study to be documented at least once, while the other two studies had some missing patient characteristics (see Fig. 1). All exposure and outcome measures were available at least once for two of the studies in our EHR (the exception was the DRO study; see Table 1). Despite similar or extended study data periods, we recruited proportionally smaller study population sizes in comparison to the original studies (see Table 2). Within the observational periods, we noticed that some patient characteristics were only documented from or up to certain points in time, so the availability of patient characteristics changed over time. Most of the structured data used for the three selected studies consisted of diagnostic and procedure codes from administrative data. The different documentation diligence and expressiveness within our EHR data provide us various opportunities to assess the data quality in the feasibility analysis.

In the feasibility analysis, we identified the data quality dimension granularity as the major data quality issue in reusing diagnosis and procedure codes for observational studies. We found patient characteristics were unavailable or could not be expressed in detail with available diagnosis and procedure codes. In addition, we noticed a poor representation of causal relationships or causal chains in codes, for instance in terms of pathophysiology, as well as the labeling of the diagnosis status, whether the diagnosis relates to an acute, chronic, suspected, recurrent or solved condition for example. These limitations in granularity leave space for different interpretations of the coding constellation, disguising and complicating the interpretation of the actual clinical course. While some hospitals may improve granularity by flagging whether a diagnosis was already present on admission [39], we could not find this information in our EHR data. Only the provenance of the data and timestamps within our EHR provided indirect clues in unclear coding constellation, which we could on occasion leverage for computed phenotypes to bypass these granularity issues.

In relation to the comparison of the patient characteristics distributions, we observed some consistent distributions of patient characteristics as well as indications of underreporting; this may represent an incomplete documentation or a realistic variation within our EHR study population. These observations may be caused by individual documentation habits, as some coders may prefer certain subsets of the wide range of coding options in diagnosis and procedure codes [28, 29, 33]. These different documentation habits may have led to the observed different study populations sizes, when relying on only one certain code set as eligibility criteria.

We believe a lack of granularity provokes incorrectness as only part of the true clinical course of a subject can be portrayed. We found the available free texts essential to the clarity and comprehensibility of patient history, and can confirm differences between diagnosis and procedure codes and free-text, as found in other studies [29, 40, 41]. Another factor that could contribute to incorrectness, is that the coding in administrative data can have multiple errors [42] and may be influenced by financial incentives [43, 44]. After the introduction of the DRGs, some studies have demonstrated an alteration of coding in administrative data [45–47]. However, it should be mentioned that improvements in coding accuracy are evident over time following the introduction of new coding system, which may relate to a coding learning curve [48].

We found that the time stamps of procedure codes reflect temporal sequence of events well, while those of some diagnosis codes did not. Understanding the patient’s individual clinical course is important to determine whether an exposure preceded an outcome or vice versa, thus enabling the distinction of causation from mere association [49]. In our case, diagnosis codes from administrative data should be seen as a summary of the patient’s hospital stay and not as an exact timeline of events. In consequence, the longer the hospital stay or the sicker the patient, the more difficult it is to disentangle the clinical course, especially when due to the lack of granularity the diagnosis or procedure code constellation could be interpretable in multiple ways. Further, an imprecise temporal sequence of diagnosis codes could make it difficult to link the data to other relevant data such as results of laboratory tests, for example for clarifying whether a particular assigned diagnosis code could be attributable to an increase in an inflammatory marker.

While it may seem expedient to replace the traditional, time-consuming manual approach of reviewing, extracting and structuring data in observational studies with the use of structured EHR data, this is a step that calls for careful consideration, insofar as ambiguities, heterogeneous documentation habits, and unclear temporal sequences and causal relationships in diagnosis and procedure codes can impede their reutilization for observational studies.

In conclusion, we interpret that unstructured data remains essential to create a comprehensible patient history [50], so that evolving tools such as natural language processing (NLP) need to be deployed to reduce the burden of manual abstraction [51]. These advanced methods, alongside their evolving practical capabilities for the computerization of EHR-based observational studies, point to various avenues for future research. A stronger focus on standardized documentation via structured documentation may also facilitate secondary use.

### Limitations

When interpreting our findings, it is vital to bear in mind that the observational studies we reviewed did not always provide clear information on the utilized data source. Table 3 critically appraises the recruited study population and the distributions of patient characteristics. The differences in the compared patient characteristics distributions were potentially attributable to the difficulties of mapping patient characteristics onto the EHR structured data, as well as to evolving medical advances. When using EHR data from longer periods of time, we cannot expect medical documentation to be entirely static over time, since it should obviously respond to scientific improvements as well as to the introduction of new definitions, classifications or regulations. More nuanced diagnostics entail revisions in the definition and classification of diseases to the end of advancing standardization and better describing disease rates and comparisons within and between populations [52, 53], which may increase or decrease the frequency of certain diagnoses. We presume, however, that such gradual developments should only have a minor impact on our findings and conclusions.

For practical reasons and due to the partial unavailability of clinical data, verification via a relative gold standard could only be applied for some patient characteristics, yet we believe the pragmatic compromise summarizes the constraints reasonably well. As our observations are drawn from a database from a single site, the entire patient history may not be included, which may ignore potential patient migration between healthcare providers (data fragmentation).

## Conclusion

The general data requirements of observational studies are not only based on routine data, but also on data solely acquired to meet investigators’ individual data needs for the study, which the EHR cannot fulfill and may require contacting former patients.

Besides the importance of patient characteristics availability, the feasibility of conducting observational studies with structured EHR data, in particular diagnosis and procedure codes from administrative data, should remain a case-by-case decision, taken in awareness of the data quality. The data quality dimension granularity is pivotal for the reuse of structured data. Currently, unstructured data such as free text is essential to complement unavailable structured data and to present a comprehensible patient history, assuring valid information on patient characteristics of interest.

## Supplementary information

**Additional file 1.** Screenshot: frequency tables, patient files, pp. 2–3. Overview of temporal data requirements for DPS/DG/DRO studies, pp. 4–6. Mapping of eligibility criteria for DPS/DG/DRO studies, pp. 7–9. Overview of available data sources, p. 10. Overview of available data in accordance with eligibility criteria applied, for DPS/DG/DRO studies, pp. 11–13. Fluctuating numbers of diagnoses per encounter, p. 14. Coding inconsistencies in chronic diseases and lifestyle factors, p. 15. Overview of eligible radiochemotherapy protocols, p. 16. Overview of assigned ICD:C25 throughout the encounters per patient ID, p. 17.

## Data Availability

Data access required authorization from the departments of Plastic and Hand Surgery, Gastroenterology and Radiation Oncology, which was obtained for the current study only. The study data is therefore not publicly available. Please direct enquiries to the Chair of Medical Informatics at Friedrich-Alexander-Universität Erlangen-Nürnberg.

## Abbreviations

ATC: Anatomical Therapeutic Chemical
CSV: Comma-Separated Values
DRG: Diagnosis Related Groups
EHR: Electronic Health Record
ICD10-GM: International Classification of Diseases 10, German Modification
ICPM: International Classification of Procedures in Medicine
MeSH: Medical Subject Headings
OPS: Operationen- und Prozedurenschlüssel
XML: Extensible Markup Language
XSLT: Extensible Stylesheet Language
