# Supplementary material for: Feasibility analysis of conducting observational studies with the electronic health record
Source: BMC Med Inform Decis Mak. 2019 Oct 28;19:202. doi: 10.1186/s12911-019-0939-0 (PMC6819452; doi:10.1186/s12911-019-0939-0)
Supplement: Supplementary file 1 — Additional file 1. Screenshot: frequency tables, patient files, pp. 2–3. Overview of temporal data requirements for DPS/DG/DRO studies, pp. 4–6. Mapping of eligibility criteria for DPS/DG/DRO studies, pp. 7–9. Overview of available data sources, p. 10. Overview of available data in accordance with eligibility criteria applied, for DPS/DG/DRO studies, pp. 11–13. Fluctuating numbers of diagnoses per encounter, p. 14. Coding inconsistencies in chronic diseases and lifestyle factors, p. 15. Overview of eligible radiochemotherapy protocols, p. 16. Overview of assigned ICD:C25 throughout the encounters per patient ID, p. 17. [file 12911_2019_939_MOESM1_ESM.pdf]

Supplementary Material

Screenshot: Frequency tables

Tabellen Diagramme Patientenakten

Universitätsklinikum Erlangen

Filter: Bösartige Neubildung des Pankreas

ICD

- Hauptdiagnosen
- Nebendiagnosen
- Diabetes mellitus
- Bösartige Neubildung des Pankreas
- Bösartige Neubildungen

OPS

- Strahlentherapie, nuklearmedizinische Therapie und Chemotherapie
- Zytostatische Chemotherapie, Immuntherapie und antiretrovirale Therapie
- Transfusion von Blutzellen
- Operationen an Gallenblase und Gallenwegen

ATC

- Antineoplasitsche Mittel

Sonstige

- Geschlecht
- Aufnahmealter

Zusammenführen von: ---

Kode	Kapitel	Gruppe	Kategorie	Subkategorie 1	Subkategorie 2	Anzahl	%
C25.0 (HD)	C00-C97	C15-C26: Bösartige Neubildungen der Verdauungsorgane	Bösartige Neubildung des Pankreas	Pankreaskopf	NA	9	64.3
C25.8 (HD)	C00-C97	C15-C26: Bösartige Neubildungen der Verdauungsorgane	Bösartige Neubildung des Pankreas	Pankreas, mehrere Teilbereiche überlappend	NA	7	50
C25.8 (ND)	C00-C97	C15-C26: Bösartige Neubildungen der Verdauungsorgane	Bösartige Neubildung des Pankreas	Pankreas, mehrere Teilbereiche überlappend	NA	7	50
C25.9 (HD)	C00-C97	C15-C26: Bösartige Neubildungen der Verdauungsorgane	Bösartige Neubildung des Pankreas	Pankreas, nicht näher bezeichnet	NA	6	42.9
C25.9 (ND)	C00-C97	C15-C26: Bösartige Neubildungen der Verdauungsorgane	Bösartige Neubildung des Pankreas	Pankreas, nicht näher bezeichnet	NA	6	42.9
C25.0 (ND)	C00-C97	C15-C26: Bösartige Neubildungen der Verdauungsorgane	Bösartige Neubildung des Pankreas	Pankreaskopf	NA	4	28.6
C25.1 (HD)	C00-C97	C15-C26: Bösartige Neubildungen der Verdauungsorgane	Bösartige Neubildung des Pankreas	Pankreaskörper	NA	1	7.1
C25.7 (HD)	C00-C97	C15-C26: Bösartige Neubildungen der Verdauungsorgane	Bösartige Neubildung des Pankreas	Sonstige Teile des Pankreas	NA	1	7.1

Übersicht: ICD

Übersicht: OPS

Übersicht: ATC

Übersicht: Labor

Übersicht: DRG

Übersicht: Sonstige

Screenshot: Patient files

Tabellen Diagramme Patientenakten

Universitätsklinikum Erlangen

Patient ID: 3

Geburtstag:
Geschlecht:

Suche...

Encounter
757
07.06.2008 - 07.06.2008
Patient ID: 3
ambulant

Verlauf

DEM

DEM|GESCHLECHT:M - männlich
DEM|AUFNALTER:44 - 44 Jahre

ICD

C25.8 (ND) Bösartige Neubildung: Pankreas, mehrere Teilbereiche überlappend - ST Station RT 52 35823 (ST52) - 2008-06-07 19:04:02

Labor

Code	Name	Wert	Flag	Einheit	Datum
LAB:6KREA	Kreatinin	0.58	@	mg/dl	2008-06-18 17:46:10
LAB:6KREC	Kreatinin-Clearance	162	H	ml/min	2008-06-18 17:46:10
LAB:6UKRE	U-Kreatinin	85.5	@	mg/dl	2008-06-18 17:46:10

Encounter
1130
18.06.2008 - 22.06.2008
Patient ID: 3
teilstationär

Verlauf

DEM

DEM|GESCHLECHT:M - männlich
DEM|AUFNALTER:44 - 44 Jahre

ICD

C25.0 (HD) Bösartige Neubildung: Pankreaskopf - ST 52 (TagesKlinik) 05823 (STTAG) - 2008-06-18 15:12:00

OPS

8-529.8 - Bestrahlungsplanung für perkutane Bestrahlung und Brachytherapie: Bestrahlungsplanung für perkutane Bestrahlung, mit individueller Dosisplanung - ST 3D-Bestrahlungsplanung (STSTD) - 2008-06-18 17:56:17
8-528.9 - Bestrahlungssimulation für externe Bestrahlung und Brachytherapie: Feldfestlegung mit Simulator, mit 3D-Plan - ST 3D-Bestrahlungsplanung (STSTD) - 2008-06-22 17:10:56
8-527.8 - Konstruktion und Anpassung von Fixations- und Behandlungshilfen bei Strahlentherapie: Individuelle Blöcke oder Viellamellenkollimator (MLC) - ST 3D-Bestrahlungsplanung (STSTD) - 2008-06-18 17:56:17
3-222 - Computertomographie des Thorax mit Kontrastmittel - ST Computertomographie (STCT) - 2008-06-19 17:56:26
3-225 - Computertomographie des Abdomens mit Kontrastmittel - ST Computertomographie (STCT) - 2008-06-19 17:56:26
8-528.9 - Bestrahlungssimulation für externe Bestrahlung und Brachytherapie: Feldfestlegung mit Simulator, mit 3D-Plan - ST 3D-Bestrahlungsplanung (STSTD) - 2008-06-18 17:56:17

Labor

Code	Name	Wert	Flag	Einheit	Datum
LAB:6ALBU	Albumin	40.4	@	g/l	2008-06-25 19:06:10
LAB:6APO	Alk. Phosphatase	168	H	U/l	2008-06-25 19:06:10

Overview of the temporal data requirements - DPS Study

Past

Disease

Initial or recurrence diagnosis

Dupuytren‘s contracture

Assessment

Treatment

One encounter

Assessment

- **Demography**
  - Sex
  - Age
- **Lifestyle**
- **Comorbidities**
- **Affected Hand**
- **Affected Digit**
- **Classifications**
  - Iselin

Treatment

- **Operation**
  - Technique
  - Simultaneous
- **Complications**

Present

Observational Study

Inclusion criteria

- Dupuytren‘s contracture
- Operation

Exclusion criteria

- Outpatient cases

Involved departments

Assessment	Plastic surgery
Treatment	Plastic surgery

Attributes of interest

Overview of the temporal data requirements - DG Study

Past			Present
**Disease**	**Assessment**	**Treatment**	**Observational Study**

One encounter

Initial or recurrence diagnosis

Esophageal stenosis

Demography
- Sex
- Age

Stenosis
- Length
- Location

Dilatation
- Type: Bougination/Balloon dilatation
- Indications

Initial or recurrence diagnosis

Perforation

- Clinic
- Laboratory
- Size
- Location

- Management
- Death

Inclusion criteria

- Esophageal stenosis
- Bougination
- Balloon dilatation

Involved departments

**Assessment**	Gastroenterology<br>Radiology
**Treatment**	Gastroenterology<br>Surgery

Attributes of interest

Overview of the temporal data requirements - DRO Study

Past

Disease

Initial diagnosis

Locally advanced pancreatic cancer

Assessment

One/multiple encounter

Classifications
- ECOG
- Grading
- TNM
- UICC

Tumor
- Location
- Diagnosed by operation or biopsy

Demography
- Sex
- Age

Treatment

Multiple encounter

Radiotherapy
- Dose
- Administration time
- Cause of adjustment

Chemotherapy
- Dose
- Administration time
- Cause of adjustment

Classifications
- ECOG
- TNM
- UICC
- Common toxicity criteria

BMI

Follow up

Multiple encounter

Classifications
- ECOG
- TNM
- UICC
- Common toxicity criteria

BMI

Death

Present

Observational Study

Inclusion criteria

- Locally advanced pancreatic cancer (LAPC)
- Ductal adenocarinoma
- Radiotherapy
- Chemotherapy (Gemcitabine and Cisplatin, 5-Fluorouracil and Mitomycin C)

Involved departments

Assessment	Gastroenterology<br>Surgery<br>Radiology
Treatment	Radiation Oncology
Follow up	Radiation Oncology<br>Radiology

Attributes of interest

Mapping of eligibility criteria - DPS Study

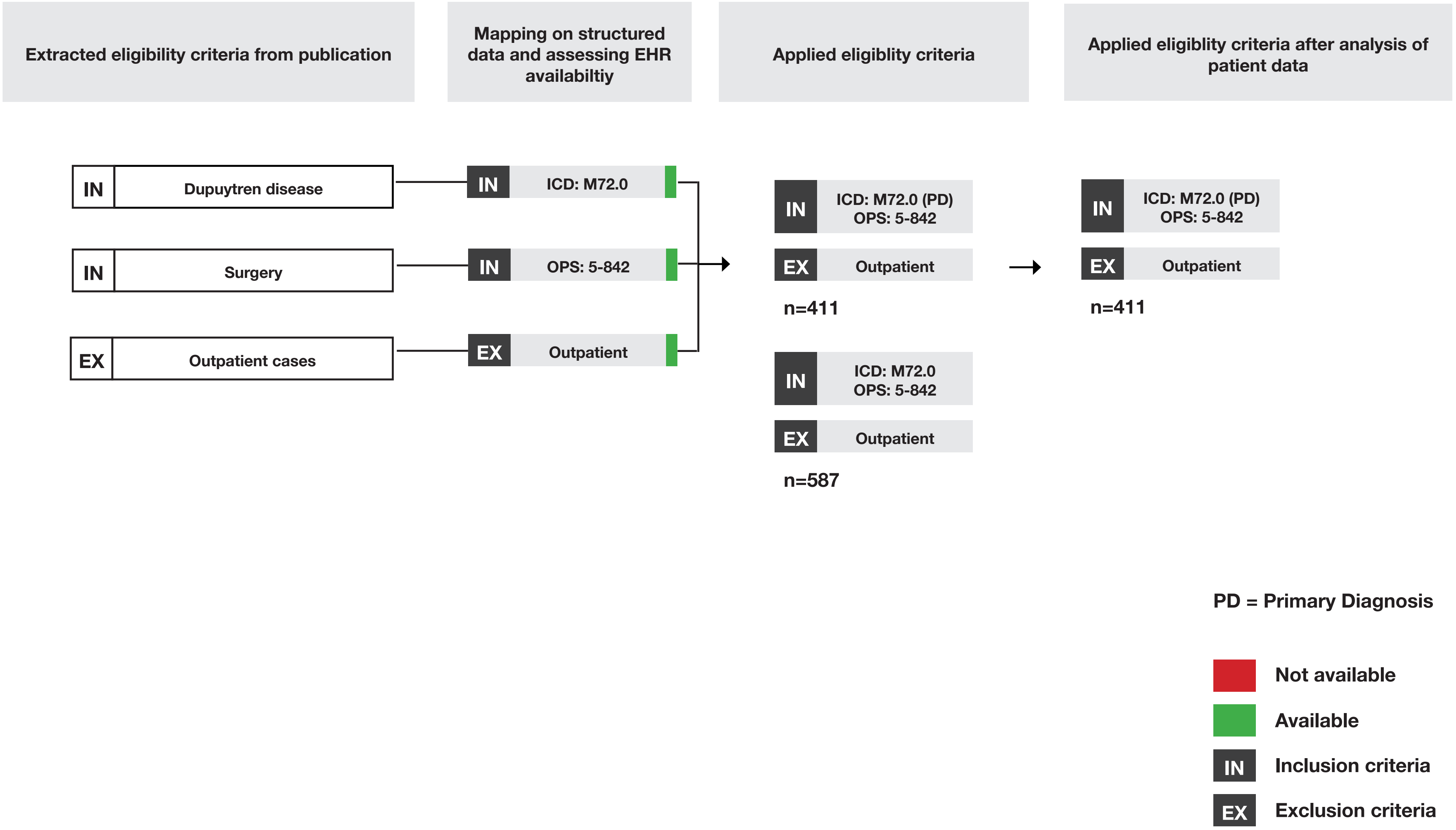

Mapping of eligibility criteria - DG Study

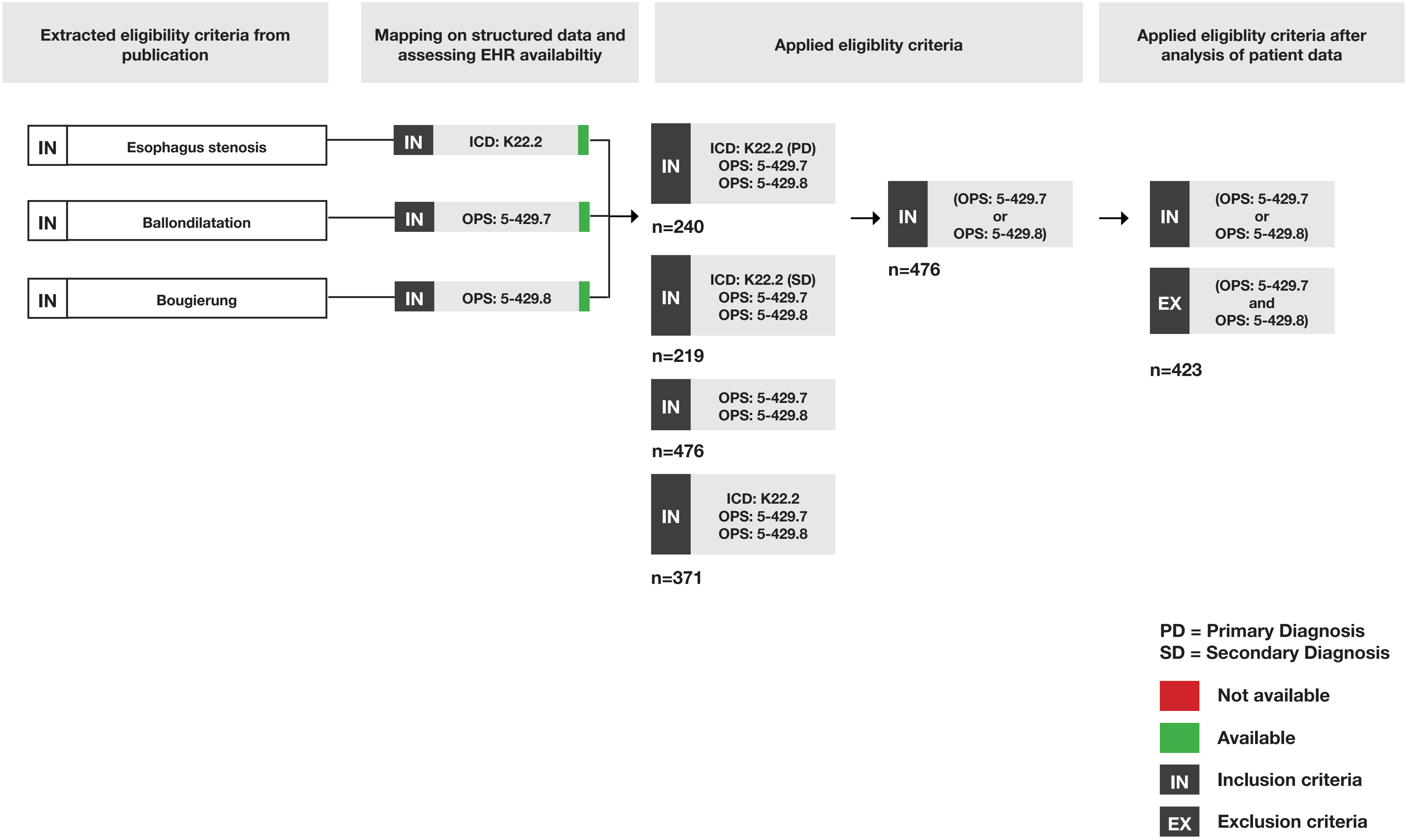

Mapping of eligibility criteria - DRO Study

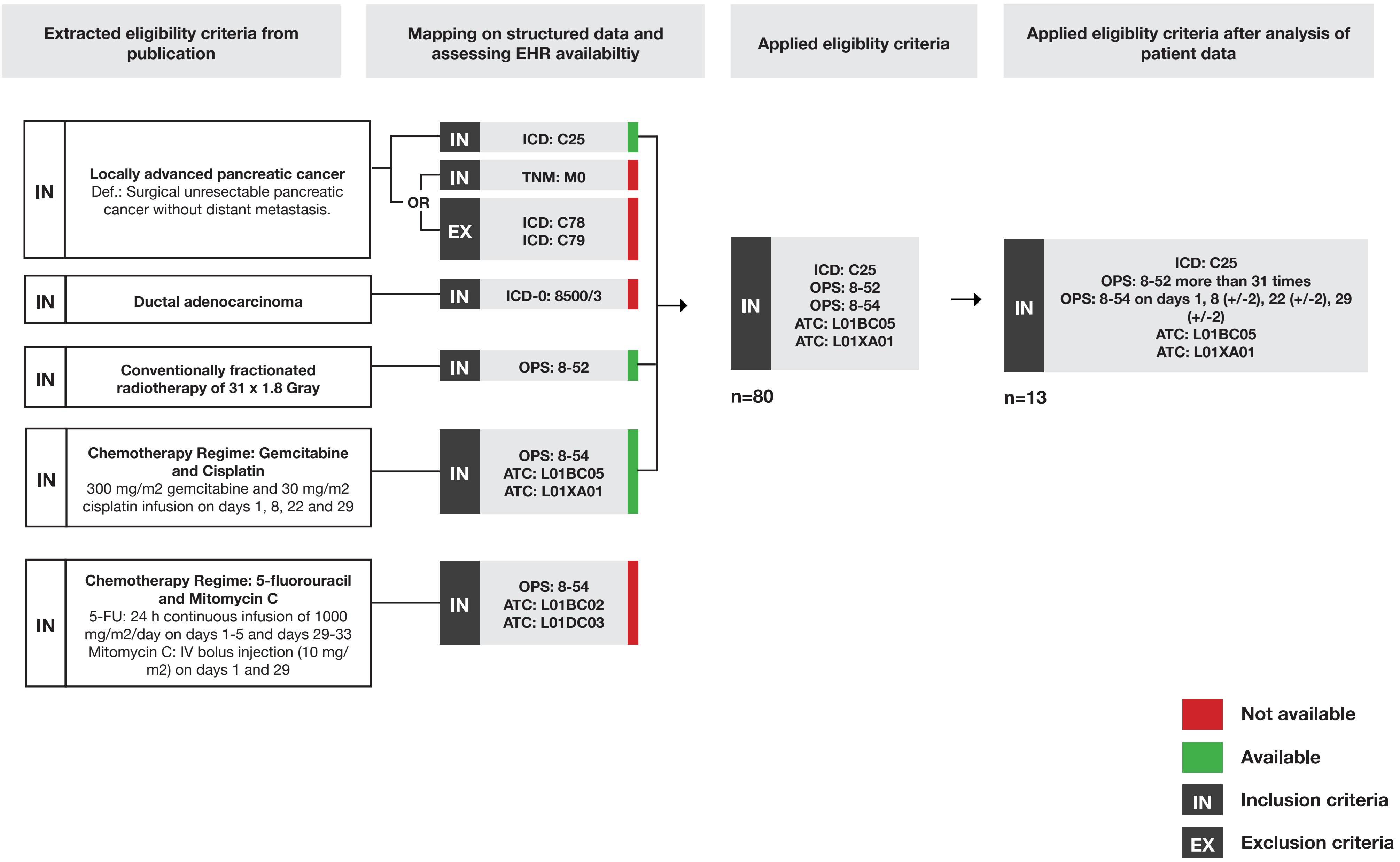

Overview of available data sources in the EHR

Administrative Data
**Structured Data**
Date of birth
Sex
Admission date
Discharge date
Diagnostic codes (ICD), primary and secondary diagnoses, timestamps, data provenance
Procedure codes (OPS), timestamps, data provenance
Date of death

Clinical Data
**Structured Data**
Laboratory
Medication (ATC)
**Unstructured data**
Pathology reports
Operations notes

Registry Data
**Structured data**
ICD-O-3

Overview of available data in accordance with eligibility criteria applied: DPS Study

Applied eligibility criteria

Inclusion
1. ICD: M72.0 (HD)
2. OPS: 5-842

Exclusion
1. Outpatient

EHR → **i2b2**

Overall data period: Nov 1999 - Sep 2016

Administrative Data		
**Structured Data**		
**Attributes**	**No. (%)**	**Average per encounter**
Sex	411 (100)	-
Date of Birth	411 (100)	-
Diagnosis codes (ICD 10-GM)<br>Period: Nov 1999 - Sep 2016	411 (100)	1
Procedure codes (OPS)<br>Period: Nov 1999 - Sep 2016	411 (100)	1
**Clinical Data**		
**Structured Data**		
**Attributes**	**No. (%)**	**Average per encounter**
Laboratory<br>Period: Jan 2001 - Dec 2012	185 (45)	0.40
**Unstructured Data**		
**Attributes**	**No. (%)**	**Average per encounter**
Pathology Reports<br>Period: May 2000 - May 2016	313 (76.2)	0.71
Operation Notes<br>Period: Nov 2000 - Sep 2016	373 (90.8)	0.91

Description
Avaliability of attributes per patient and the average availability per encounter.

Overview of available data in accordance with eligibility criteria applied: DG Study

EHR	i2b2	i2b2
	**Applied eligibility criteria**<br>**Inclusion**<br>1. OPS: 5-429.7 or OPS: 5-429.8	**Applied eligibility criteria**<br>**Inclusion**<br>1. OPS: 5-429.7 or OPS: 5-429.8<br>**Exclusion**<br>1. OPS: 5-429.7 and OPS: 5-429.8

Overall data period: Jan 2000 - Sep 2016

Administrative Data		
**Structured Data**		
**Attributes**	**No. (%)**	**Average per encounter**
Sex	476 (100)	-
Date of Birth	476 (100)	-
Date of Death	107 (22.5)	-
Diagnosis codes (ICD 10-GM)<br>Period: Jun 2001 - Sep 2016	454 (95.4)	0.96
Procedure codes (OPS)<br>Period: Dec 2000 - Sep 2016	476 (100)	1
**Clinical Data**		
**Structured Data**		
**Attributes**	**No. (%)**	**Average per encounter**
Laboratory<br>Period: Dec 2002 - Jan 2012	255 (53.6)	0.45
**Unstructured Data**		
**Attributes**	**No. (%)**	**Average per encounter**
Pathology Reports<br>Period: Sep 2001 - Jun 2016	186 (39.1)	0.21
Operation Notes<br>Period: Jan 2001 - Aug 2016	41 (8.6)	0.03

Overall data period: Dec 2000 - Sep 2016

Administrative Data		
**Structured Data**		
**Attributes**	**No. (%)**	**Average per encounter**
Sex	423 (100)	-
Date of Birth	423 (100)	-
Date of Death	93 (21.9)	-
Diagnosis codes (ICD 10-GM)<br>Period: Jun 2001 - Sep 2016	404 (95.5)	0.96
Procedure codes (OPS)<br>Period: Dec 2000 - Sep 2016	423 (100)	1
**Clinical Data**		
**Structured Data**		
**Attributes**	**No. (%)**	**Average per encounter**
Laboratory<br>Period: Dec 2002 - Jan 2012	213 (50.3)	0.44
**Unstructured Data**		
**Attributes**	**No. (%)**	**Average per encounter**
Pathology Reports<br>Period: Sep 2001 - Jun 2016	161 (38.1)	0.25
Operation Notes<br>Period: Jan 2001 - Aug 2016	35 (8.3)	0.04

Description
Avaliability of attributes per patient and the average availability per encounter.

Overview of available data in accordance with eligibility criteria applied: DRO Study

EHR → i2b2 → i2b2 → i2b2

i2b2

Applied eligibility criteria

1. ICD: C25
2. OPS: 8-52
3. OPS: 8-54

Overall data period: Jan 2000 - Jun 2015

Administrative Data		
**Structured Data**		
**Attributes**	**No. (%)**	**Average per encounter**
Sex	243 (100)	-
Date of Birth	243 (100)	-
Date of Death	49 (20.2)	-
Diagnosis codes (ICD 10-GM)<br>Period: Jan 2000 - Sep 2015	243 (100)	0.97
Procedure codes (OPS)<br>Period: Oct 2002 - Sep 2015	243 (100)	0.80
**Clinical Data**		
**Structured Data**		
**Attributes**	**No. (%)**	**Average per encounter**
Laboratory<br>Period: Aug 2003 - Jan 2012	181 (74.4)	0.38
Medication (ATC)<br>Period: Jan 2004 - Sep 2015	186 (76.5)	0.26
**Unstructured Data**		
**Attributes**	**No. (%)**	**Average per encounter**
Pathology Reports<br>Period: Oct 2002 - Jun 2015	40 (16.5)	0.02
Operation Notes<br>Period: Jan 2008 - Nov 2014	76 (31.3)	0.03
**Registry Data**		
**Structured Data**		
**Attributes**	**No. (%)**	**Average per encounter**
ICD-O-3<br>Period: Jun 2015	1 (0.4)	-

i2b2

Applied eligibility criteria

1. ICD: C25
2. OPS: 8-52
3. OPS: 8-54
4. ATC: L01BC05
5. ATC: L01XA01

Overall data period: Oct 2007 - Sep 2015

Administrative Data		
**Structured Data**		
**Attributes**	**No. (%)**	**Average per encounter**
Sex	80 (100)	-
Date of Birth	80 (100)	-
Date of Death	18 (22.5)	-
Diagnosis codes (ICD 10-GM)<br>Period: Oct 2007 - Sep 2015	80 (100)	0.995
Procedure codes (OPS)<br>Period: Oct 2007 - Jul 2015	80 (100)	0.87
**Clinical Data**		
**Structured Data**		
**Attributes**	**No. (%)**	**Average per encounter**
Laboratory<br>Period: Oct 2010 - Jan 2012	69 (86.3)	0.47
Medication (ATC)<br>Period: Nov 2007 - Nov 2014	80 (100)	0.34
**Unstructured Data**		
**Attributes**	**No. (%)**	**Average per encounter**
Pathology Reports<br>Period: Apr 2008 - Apr 2013	15 (18.8)	0.02
Operation Notes<br>Period: Jun 2008 - May 2014	23 (29)	0.02
**Registry Data**		
**Structured Data**		
**Attributes**	**No. (%)**	**Average per encounter**
ICD-O-3<br>Period: -	-	-

i2b2

Applied eligibility criteria

1. ICD: C25
2. OPS: 8-52 more than 31 times
3. OPS: 8-54 on days 1, 8, 22, 29 (+/-2)
4. ATC: L01BC05
5. ATC: L01XA01

Overall data period: Jan 2008 - May 2015

Administrative Data		
**Structured Data**		
**Attributes**	**No. (%)**	**Average per encounter**
Sex	13 (100)	-
Date of Birth	13 (100)	-
Date of Death	3 (21.4)	-
Diagnosis codes (ICD 10-GM)<br>Perdio: Jan 2008 - May 2015	13 (100)	1
Procedure codes (OPS)<br>Perdio: Jan 2008 - May 2015	13 (100)	0.86
**Clinical Data**		
**Structured Data**		
**Attributes**	**No. (%)**	**Average per encounter**
Laboratory<br>Period: Jan 2008 - Nov 2011	11 (84.6)	0.47
Medication (ATC)<br>Period: Jan 2008 - Oct 2012	13 (100)	0.27
**Unstructured Data**		
**Attributes**	**No. (%)**	**Average per encounter**
Pathology Reports<br>Period: Apr 2008 - Mar 2013	3 (23.1)	0.02
Operation Notes<br>Period: Aug 2008 - Dec 2009	3 (23.1)	0.02
**Registry Data**		
**Structured Data**		
**Attributes**	**No. (%)**	**Average per encounter**
ICD-O-3<br>Period: -	-	-

Description
Avaliability of attributes per patient and the average availability per encounter.

Fluctuating numbers of diagnoses per encounter

DPS Study

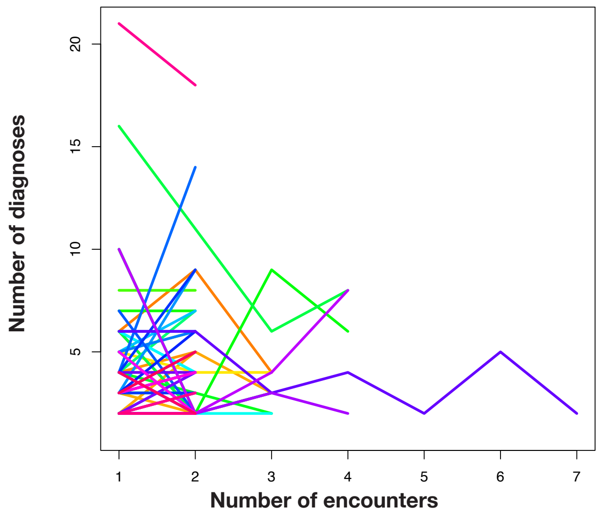

DG Study

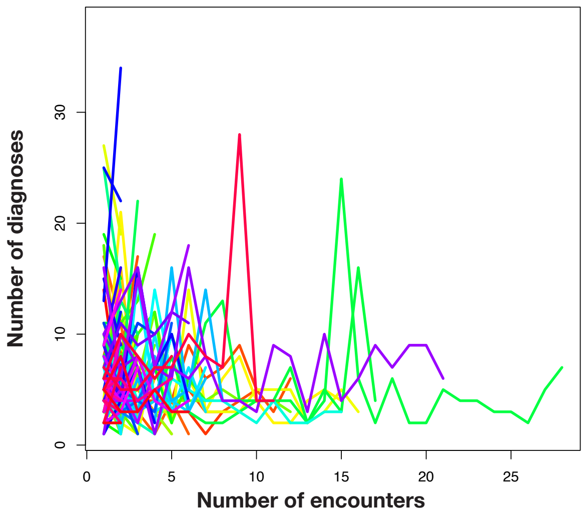

DRO Study

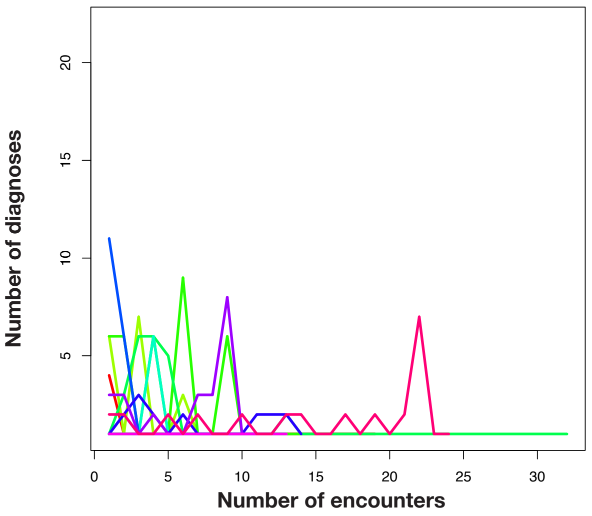

Potential coding inconsistencies in chronic diseases and lifestyle factors - DPS Study

Chronic diseases

E10-E14 Diabetes mellitus
3 of 25 patient show inconsistency:

Patient ID	Encounter	Consistency
**1182**	**6370**	Coded
	**6400**	Not coded
**985**	**6585**	Not coded
	**6758**	Coded
**994**	**6760**	Not coded
	**6813**	Coded

G40.- Epilepsy
2 of 5 patient show inconsistency:

Patient ID	Encounter	Consistency
**1084**	**6310**	Coded
	**6461**	Coded
	**6601**	Coded
	**6634**	Not coded
	**6648**	Not coded
	**6723**	Not coded
	**6857**	Not coded
**902**	**6388**	Not coded
	**6539**	Coded
	**6628**	Not coded

Lifestyle

F10.- Mental and behavioural disorders due to use of alcohol
1 of 11 patient show inconsistency:

Patient ID	Encounter	Consistency
**775**	**6618**	Not coded
	**6722**	Not coded
	**6771**	Coded

F17.- Mental and behavioural disorders due to use of tobacco
2 of 4 patient show inconsistency:

Patient ID	Encounter	Consistency
**784**	**6466**	Coded
	**6603**	Not coded
**1112**	**6358**	Not coded
	**6683**	Not coded
	**6812**	Not coded
	**6862**	Coded

Overview of the eligible radiochemotherapies protocols - DRO Study

OPS:8-54: Chemotherapy

Patient ID	CODE	T1	T2	T3	T4	N
3	OPS:8-54	0	8	23	29	4
14	OPS:8-54	0	8	23	30	4
27	OPS:8-54	0	8	22	29	4
33	OPS:8-54	0	7	22	29	4
76	OPS:8-54	0	7	23	28	4
82	OPS:8-54	0	8	22	30	4
86	OPS:8-54	0	7	20	28	4
106	OPS:8-54	0	8	22	29	4
163	OPS:8-54	0	8	22	29	4
169	OPS:8-54	0	7	21	28	4
195	OPS:8-54	0	10	23	28	4
205	OPS:8-54	0	8	24	31	4
223	OPS:8-54	0	9	23	29	4

OPS:8-522: Radiotherapy

Patient ID	CODE	T1	T2	T3	T4	T5	T6	T7	T8	T9	T10	T11	T12	T13	T14	T15	T16	T17	T18	T19	T20	T21	T22	T23	T24	T25	T26	T27	T28	T29	T30	T31	T32	N
3	OPS:8-522	0	2	3	4	5	8	9	10	11	12	15	16	17	19	22	23	24	25	26	29	30	NA	NA	NA	NA	NA	NA	NA	NA	NA	NA	NA	21
14	OPS:8-522	0	2	3	6	7	8	9	10	13	14	15	16	17	20	22	23	24	27	28	29	30	31	34	35	36	37	38	41	42	43	44	NA	31
27	OPS:8-522	0	2	3	6	8	9	10	13	14	15	16	17	21	22	23	24	27	28	29	30	31	34	35	36	37	38	41	42	NA	NA	NA	NA	28
33	OPS:8-522	0	2	3	4	5	8	9	10	11	12	15	17	18	18	19	22	23	24	25	26	31	32	34	36	37	38	40	43	44	45	NA	NA	30
76	OPS:8-522	0	2	3	4	5	8	9	10	11	12	15	16	17	18	19	22	23	23	24	25	26	29	30	31	32	33	36	37	38	39	43	44	32
82	OPS:8-522	0	2	3	4	5	8	9	10	11	12	15	16	17	18	19	22	23	24	25	26	29	30	31	32	33	34	36	37	43	44	NA	NA	30
86	OPS:8-522	0	2	3	4	5	9	10	11	12	13	15	16	17	22	23	24	25	26	29	31	33	36	37	38	39	40	43	44	45	46	NA	NA	30
106	OPS:8-522	0	1	2	3	4	8	9	10	11	11	15	15	16	17	18	21	22	23	24	25	28	29	30	31	32	35	36	37	38	39	42	43	32
163	OPS:8-522	0	9	10	11	12	15	16	17	18	19	22	23	24	25	26	29	30	31	32	33	36	37	38	39	40	43	44	NA	NA	NA	NA	NA	27
169	OPS:8-522	0	2	3	4	5	8	9	10	11	12	15	16	17	18	19	22	23	24	25	26	29	30	31	32	33	37	38	39	40	43	44	NA	31
195	OPS:8-522	0	2	4	6	7	7	8	11	12	13	14	15	18	19	20	21	23	25	26	27	28	29	33	33	34	35	36	39	40	41	42	NA	31
205	OPS:8-522	0	2	3	4	5	8	9	10	11	12	15	16	17	18	19	22	23	24	25	26	29	30	31	32	33	36	37	38	39	39	40	NA	31
223	OPS:8-522	0	1	2	3	6	8	8	9	10	13	14	15	17	20	22	22	23	24	27	29	29	30	31	34	35	36	37	38	41	42	43	NA	31

Overview of assigned ICD:C25 throughout the encounters per patient ID - DRO Study

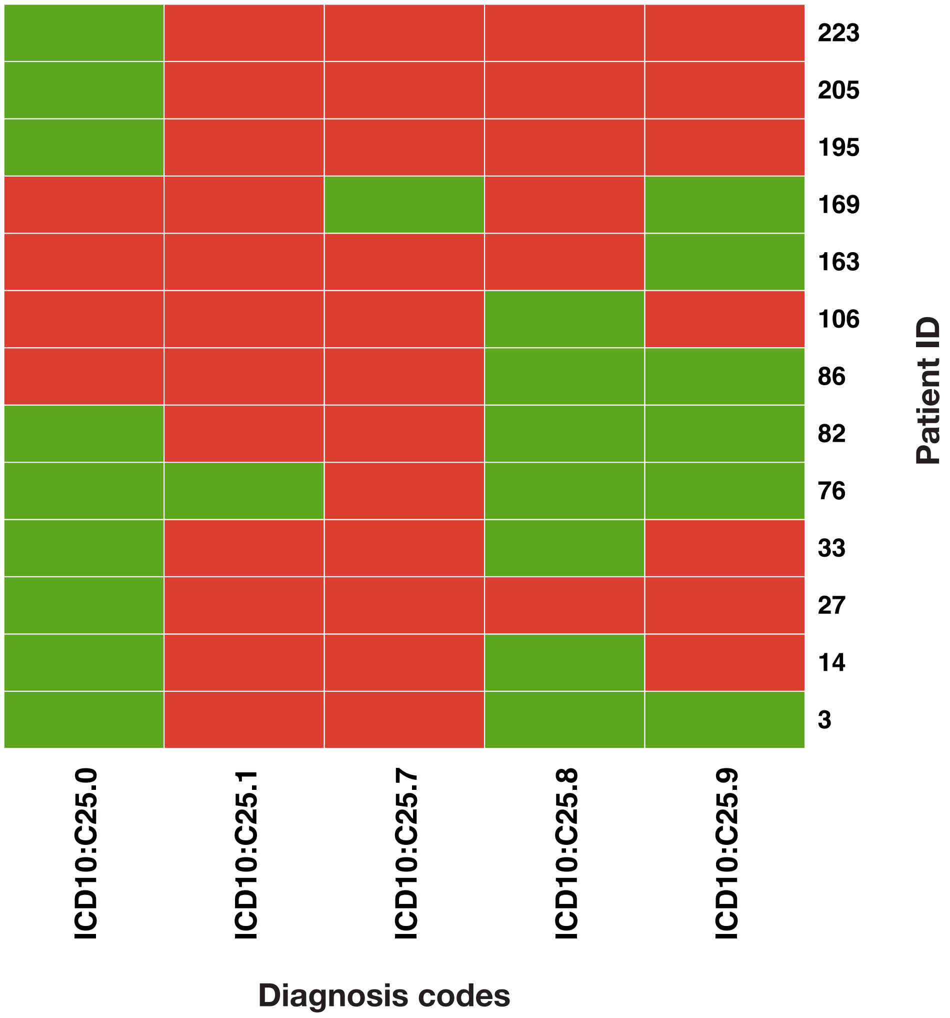

Malignant neoplasm of pancreas

C25.0 Head of pancreas
C25.1 Body of pancreas
C25.7 Other parts of pancreas (Neck of pancreas)
C25.8 Overlapping lesion of pancreas
C25.9 Pancreas, unspecified
